# Design and Application of Mesoporous Catalysts for Liquid-Phase Furfural Hydrogenation

**DOI:** 10.3390/molecules30061270

**Published:** 2025-03-12

**Authors:** Hyeongeon Lee, Shinjae Lee, Kwangjin An

**Affiliations:** School of Energy and Chemical Engineering, Ulsan National Institute of Science and Technology (UNIST), Ulsan 44919, Republic of Korea; gusrjs7@unist.ac.kr (H.L.); lsj473290@unist.ac.kr (S.L.)

**Keywords:** mesoporous, catalyst, furfural, hydrogenation, biomass upgrading

## Abstract

Furfural (FAL), a platform molecule derived from biomass through acid-catalyzed processes, holds significant potential for producing various value-added chemicals. Its unique chemical structure, comprising a furan ring and an aldehyde functional group, enables diverse transformation pathways to yield products such as furfuryl alcohol, furan, tetrahydrofuran, and other industrially relevant compounds. Consequently, optimizing catalytic processes for FAL conversion has garnered substantial attention, particularly in selectivity and efficiency. The liquid-phase hydrogenation of FAL has demonstrated advantages, including enhanced catalyst stability and higher product yields. Among the catalysts investigated, mesoporous materials have emerged as promising candidates because of their high surface area, tunable pore structure, and ability to support highly dispersed active sites. These attributes are critical for maximizing the catalytic performance across various reactions, including FAL hydrogenation. This review provides a comprehensive overview of recent advances in mesoporous catalyst design for FAL hydrogenation, focusing on synthesis strategies, metal dispersion control, and structural optimization to enhance catalytic performance. It explores noble metal-based catalysts, particularly highly dispersed Pd systems, as well as transition-metal-based alternatives such as Co-, Cu-, and Ni-based mesoporous catalysts, highlighting their electronic structure, bimetallic interactions, and active site properties. Additionally, metal–organic frameworks are introduced as both catalysts and precursors for thermally derived materials. Finally, key challenges that require further investigation are discussed, including catalyst stability, deactivation mechanisms, strategies to reduce reliance on external hydrogen sources, and the impact of solvent effects on product selectivity. By integrating these insights, this review provides a comprehensive perspective on the development of efficient and sustainable catalytic systems for biomass valorization.

## 1. Introduction

Fossil fuels continue to dominate as the primary global sources of energy and chemicals [1,2,3,4]. However, the pressing need to mitigate environmental challenges and reduce reliance on fossil fuel resources has intensified efforts to identify sustainable alternatives. Renewable energy sources such as solar, wind, hydro, and biomass are promising solutions for combating climate change while facilitating the production of chemicals and fuels in an environmentally friendly manner [5,6,7]. The increasing demand for eco-friendly products has fueled interest in transitioning from petroleum-based chemicals to biomass-derived alternatives. Biomass, as a renewable and carbon-neutral energy source, offers significant environmental advantages, including reduced greenhouse gas emissions and the alleviation of fossil resource depletion [2,8,9,10]. Notably, the CO_2_ released during biomass conversion was reabsorbed during regrowth, forming a closed carbon cycle. Although edible biomass has been explored for chemical and fuel production, its use raises concerns about food security and provides only limited greenhouse gas reduction [11]. In contrast, nonedible biomass, particularly lignocellulosic biomass, has emerged as a sustainable and ecofriendly alternative [7,12,13,14]. Although its commercialization is challenging because of its complex processes, lignocellulosic biomass has great potential for sustainable conversion routes. Various thermochemical, biochemical, and chemocatalytic approaches have been developed to convert lignocellulosic biomass into valuable chemicals [7,12,13]. Among these, chemocatalytic conversion using solid catalysts has gained attention because of its efficiency and eco-friendliness. However, the diverse functional groups in biomass-derived carbohydrates require intricate upgrading processes to transform them into usable fuels and chemicals. Simplifying these processes requires the development of high-performance catalysts with precise selectivity for tailoring the properties of the final products.

Biomass derivatives rich in unsaturated oxygen species represent an important class of compounds for catalytic conversion, benefiting both the energy sector and catalytic research. Oxygenated chemicals with high added value and minimal hydrogen consumption are attractive targets. Among these, furfural (FAL) stands out as a promising platform molecule for the sustainable production of fuels and chemicals [8,15,16,17,18]. Derived from the acid-catalyzed hydrolysis and dehydration of hemicellulose, FAL plays a pivotal role in biomass valorization. However, their poor storage stability due to two active functional groups necessitates further upgrades to produce stable products compatible with petroleum-derived fuels and industrial chemicals [19,20].

**Figure 1:**
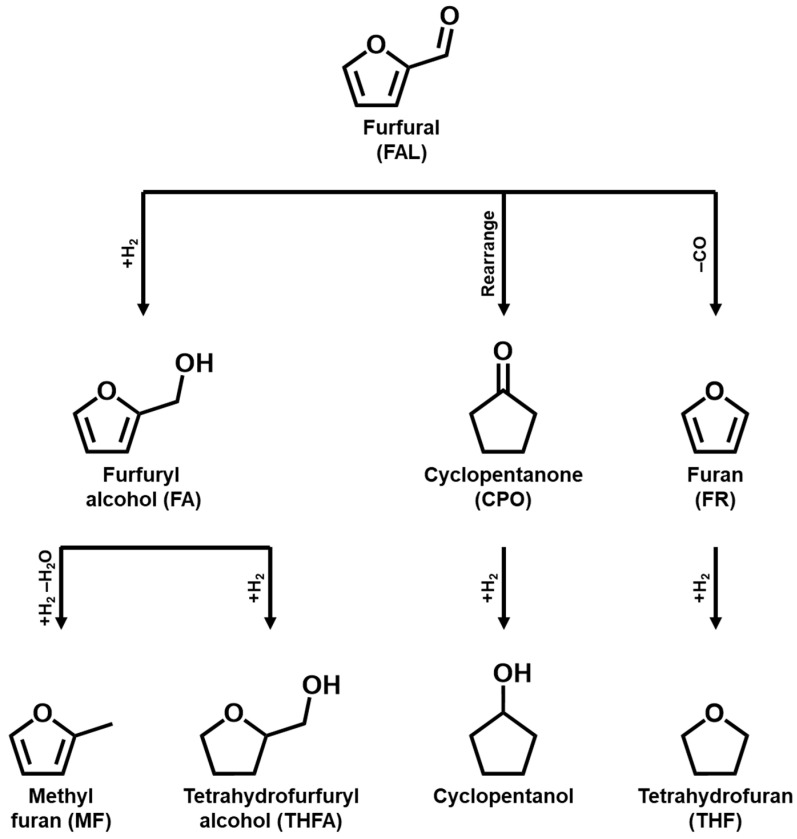
Various value-added chemicals derived from FAL through hydrogenation.

The reactivity of FAL is attributed to its furan ring and aldehyde functional groups, which enable multiple transformation pathways such as hydrogenation, rearrangement, ring opening, and decarbonylation (Figure 1) [21,22,23,24,25,26,27,28,29,30,31,32,33]. These pathways produce a range of valuable products, including furfuryl alcohol (FA), furan (FR), methylfuran (MF), tetrahydrofuran (THF), tetrahydrofurfuryl alcohol (THFA), cyclopentanone (CPO), and olefins. Each product has a distinct industrial application. For example, FA is used in resin production and as an aromatic extractor in lubricating oils [34]. In contrast, THFA serves as a green biodegradable solvent [35]. The MF, obtained via the hydrodeoxygenation of FAL, acts as a fuel additive [36]. Similarly, FR and THF, generated during decarbonylation, were employed as the solvent and fuel additive, respectively [37]. CPO, derived from FAL rearrangements, has perfumes, pesticides, and pharmaceutical applications [38]. Therefore, the development of efficient catalysts for the conversion of FAL into these valuable chemicals is crucial.

Numerous catalysts have been investigated for the hydrogenation of FAL in gas and liquid phases [39,40]. Although gas-phase reactions, often employing Cu-supported silica catalysts, can achieve high FA yields, they face challenges such as byproduct formation and polymerization, which can lead to reactor blockage and safety concerns [39]. In contrast, liquid-phase hydrogenation has shown great promise for delivering superior catalytic performances and FA yields under various conditions [40]. Heterogeneous catalysts are preferred over homogeneous catalysts for due hydrogenation because of their stability and ease of separation [12]. Supported metal catalysts, including those based on noble and transition metals, have been extensively investigated. Catalyst selection significantly influences the product distribution, as different metals exhibit unique affinities for the reaction intermediates. For example, Pd catalysts selectively produce MF and THFA at low temperatures in the aqueous phase while favoring decarbonylation to FR at higher temperatures [24,25]. Although noble metal catalysts exhibit high activity, non-precious-metal-based catalysts, such as Co, Cu, and Ni, are gaining attention owing to their cost-effectiveness, unique structures, and high dispersibility on supports [27,30]. Consequently, achieving the desired product selectivity requires careful consideration of metal selection, catalyst design, and process optimization.

This review highlights recent advancements in the design and optimization of efficient catalysts, especially mesoporous catalysts, for FAL hydrogenation. The discussion begins with an examination of mesoporous material synthesis, emphasizing their structural and textural properties that enhance catalytic performance. It then explores noble metal-based catalysis, focusing on highly dispersed Pd catalysts incorporated into mesoporous frameworks, which exhibit superior activity and stability. Transition-metal-based catalysts, including Co-, Cu-, and Ni-based mesoporous systems, are explored next, with particular attention to their tunable oxidation states, bimetallic synergies, and surface active species influencing selectivity. We also introduce metal-organic frameworks (MOFs) as a distinct class of porous materials, examining their role as both pristine catalysts and precursors for thermally derived metal-based catalysts. Finally, key challenges requiring further investigation are outlined, including catalyst stability and deactivation mechanism, strategies to reduce external hydrogen usage, and the influence of solvent on product selectivity. By integrating insights from these different catalytic systems, this review provides a comprehensive understanding of how structural design, metal dispersion, and active site accessibility contribute to the development of efficient catalysts for sustainable biomass valorization.

## 2. Synthesis of Mesoporous Materials

### 2.1. Mesoporous Materials

The use of mesoporous materials represents an effective approach for the design and optimization of efficient catalysts [25,26,27,28,29,30,31,41,42,43,44,45]. These materials are widely employed as catalysts and support owing to their desirable properties, including a high surface area, uniform and tunable pore size and shape, and the ability to adopt diverse structures and configurations. Furthermore, their highly crystalline frameworks with uniformly distributed pores make mesoporous materials particularly advantageous for investigating the relationships among structure, composition, porosity, and catalytic performance. In other words, their tailored porosities and structural properties provide valuable insights into their catalytic performance, facilitating advancements in various catalytic applications. Many reports explored various methods to synthesize mesoporous materials including silica, metal oxides, and carbons.

### 2.2. Templating Methods

Templating methods are among the well-known and well-developed approaches and are typically classified into two categories: soft-templating and hard-templating.

**Figure 2:**
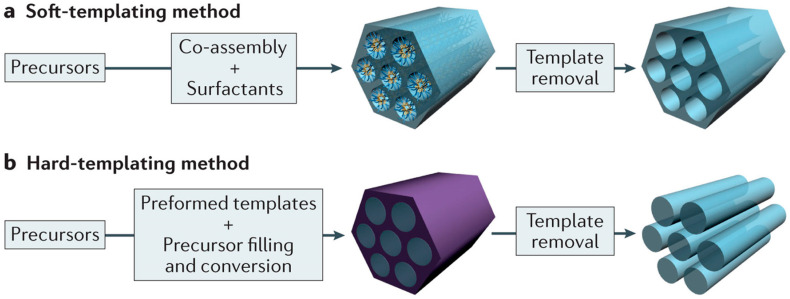
Preparation method of ordered mesoporous materials: (**a**) soft- and (**b**) hard-templating (nanocasting).

Soft-templating involves the use of surfactant micelles as templates, with mesoporous silica being a representative example synthesized through this technique (Figure 2a) [46,47]. In this process, silica precursors undergo hydrolysis around the surfactant micelles during this process, forming uniform pore structures. Subsequent calcination removes the organic templates, leaving behind ordered mesoporous silica. The synthesized mesoporous silica can be used as a support material and a sacrificial template for mesoporous metal oxides or carbons.

A widely used hard-templating method is nanocasting, which employs mesoporous silica as a template to fabricate mesoporous materials with reverse-replica structures. This technique involves infiltrating the internal pores of mesoporous silica with metal salts or carbon precursors, followed by heat treatment to create a mesoporous structure that mirrors the template (Figure 2b) [46,48,49]. The silica template was removed using NaOH or HF solution to yield the desired mesoporous material. The final structure of the resulting transition metal oxides or carbons is determined based on the pore structure of the template. Because of the flexibility of these templating methods and the broad range of accessible structures enabled by different templates, mesoporous metal oxides and carbon supports with well-ordered pores are essential for catalyst research.

### 2.3. Other Methods

Beyond templating, several alternative synthesis approaches have been explored for fabricating mesoporous materials with well-defined structures and tunable porosity. One such approach is the evaporation-induced self-assembly (EISA) method, which is closely related to the soft-templating technique [50,51]. One key difference between the two methods is the surfactant concentration. For EISA, the concentration of surfactant is low, so the surfactants do not assemble into micelles at the initial stage. Therefore, solvent evaporation is required to concentrate the surfactant and finally to form the micelle template. During the evaporation, inorganic precursors surround the outer surface of the micelle, producing an inorganic–micelle composite, which can further be transformed into mesoporous inorganic materials by thermal treatment. By adjusting the chemical and process parameters, the final structure of nanostructured materials can be controlled.

Another promising approach is reticular chemistry, which involves organic linkers and/or metal ions or clusters to create well-defined porous structures with large surface areas. Representative examples are MOFs and covalent organic frameworks [46,52]. These materials can be synthesized by various routes, including mechanochemical, diffusion, solvothermal, etc. [52,53]. For example, zeolitic imidazolate framework-67 (ZIF-67), a type of MOF, can be simply obtained by mixing and aging Co^2+^ and 2-methylimidazole solutions [54,55]. Pore sizes and surface areas of MOFs are tunable by carefully selecting the metal ions, organic linkers, and synthesis conditions.

In the following sections, we will discuss mesoporous materials synthesized using the aforementioned methods, mainly the templating approach, and their effects on FAL hydrogenation.

## 3. Noble-Metal-Based Catalysts for FAL Hydrogenation

### 3.1. Supported Pd Nanoparticle Catalysts

**Figure 3:**
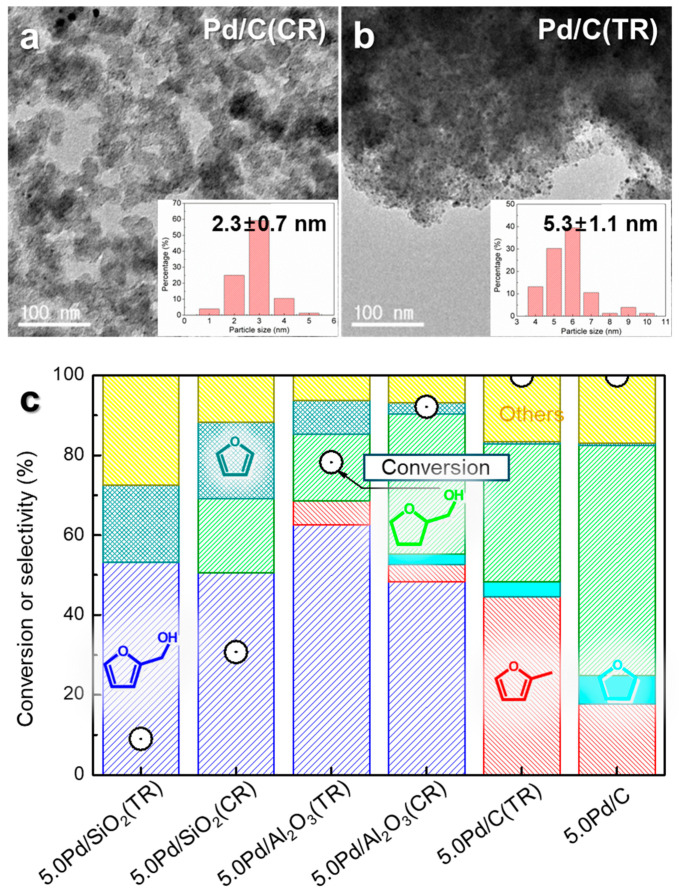
TEM images and size distribution of the supported Pd catalysts: (**a**) 5.0Pd/C (CR), (**b**) 5.0Pd/C (TR); CR: chemical reduction and TR: thermal reduction. (**c**) Hydrogenation of FAL over supported Pd catalysts.

**Figure 4:**
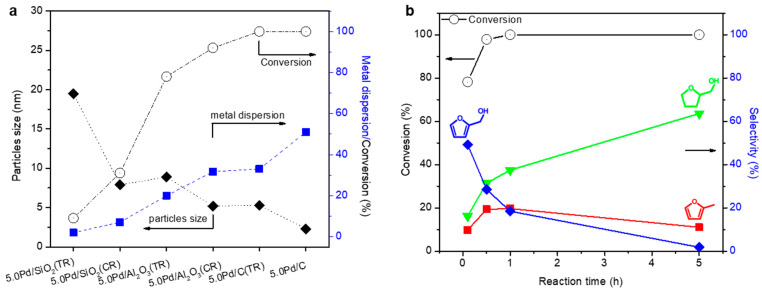
(**a**) Total conversion and of the FAL hydrogenation reaction dependent on metal dispersion and Pd particle size in the supported Pd catalysts. (**b**) Selectivity and conversion of FAL as a function of reaction time using Pd/C as a catalyst.

In noble-metal-based catalysis, achieving highly dispersed active metal species on support materials is crucial for optimizing catalytic performance. This study focuses on the preparation of supported Pd nanoparticle (NP) catalysts with well-dispersed Pd species, synthesized via chemical reduction and impregnation followed by thermal reduction (Figure 3 and Figure 4) [24]. The effectiveness of these methods in controlling the Pd NP size and metal dispersion was evaluated using supports with varying properties, including carbon, SiO_2_, and Al_2_O_3_. The sizes of the Pd NPs were characterized using X-ray diffraction (XRD) and transmission electron microscopy (TEM) (Figure 3a,b). The XRD patterns revealed broader Pd peaks for the catalysts prepared via chemical reduction compared to those prepared via thermal reduction, indicating smaller particle sizes. The TEM images confirmed this, showing narrower size distributions for chemical reduction. Among the supports tested, carbon enabled the highest Pd dispersion (2.3 and 5.3 nm), followed by Al_2_O_3_ (5.2 and 8.9 nm) and SiO_2_ (7.9 and 19.5 nm). The performances of these Pd catalysts for liquid-phase FAL hydrogenation were evaluated (Figure 3c). The Pd/C catalyst achieved 100% FAL conversion, outperforming the Pd/Al_2_O_3_ (78–92%) and Pd/SiO_2_ (9–31%) catalysts. Additionally, catalysts prepared via chemical reduction consistently demonstrated higher conversion rates than those prepared via thermal reduction, underscoring the critical role of metal dispersion. Product selectivity also varied with the support type: Pd/C produced THFA and MF as the major products, whereas Pd/SiO_2_ and Pd/Al_2_O_3_ primarily produced FA. A negative correlation was observed between the Pd particle size and FAL conversion (Figure 4a), emphasizing the catalytic benefits of the small Pd particles and the high surface area provided by the carbon supports. Notably, the highly dispersed Pd/C catalyst prepared via chemical reduction exhibited superior performance for THFA production compared to traditional supported catalysts with larger Pd particles. Reaction time studies with Pd/C further illustrate the sequential hydrogenation pathway of FAL to FA and subsequently to THFA (Figure 4b). Within 1 h, FAL conversion reached 100%, yielding 18% FA and 38% THFA. Extending the reaction time to 5 h increased the THFA yield to 64%, while reducing the FA yield to 2%. The recyclability of Pd/C was also assessed, showing no significant loss in FAL conversion over five cycles, with only a slight decrease in THFA selectivity.

### 3.2. Highly Dispersed Pd Catalysts Supported on Various Carbons

Previous research has demonstrated that Pd supported on carbon (Pd/C) catalysts outperform Al_2_O_3_- and SiO_2_-supported catalysts in FAL hydrogenation because of the higher dispersion of Pd particles on carbon [24]. Carbon-based supports are widely used in catalysis due to their excellent physical, chemical, and mechanical properties, along with high thermal and electrical conductivities [56,57]. The advent of ordered mesoporous carbons (OMCs) has enabled the design of porous carbons with tailored physicochemical properties and large surface areas. OMCs, such as the CMK series (e.g., CMK-3, CMK-5, and CMK-8), have been synthesized via nanocasting methods using silica templates such as SBA-15 and KIT-6, providing opportunities to study mass transfer and reaction dynamics as a function of pore size and structure, particularly in liquid-phase reactions, where mass transfer plays a significant role.

**Figure 5:**
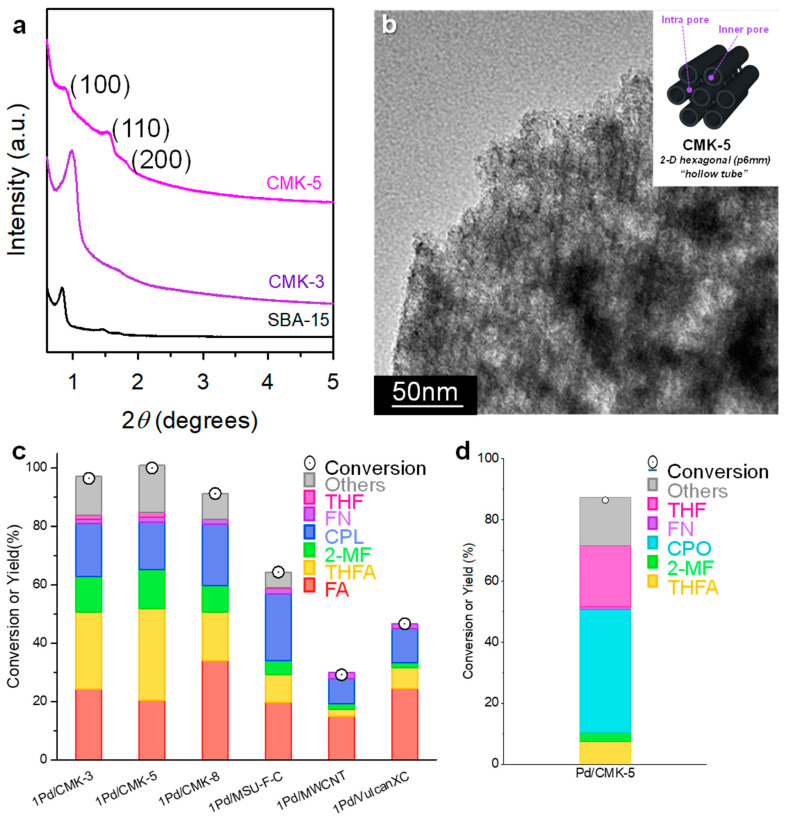
Low-angle XRD patterns of Pd/C catalysts; (**a**) SBA-15 template, CMK-3, and CMK-5. (**b**) TEM images of synthesized Pd/CMK-5 catalyst. (**c**) Conversion and yield of FAL hydrogenation using Pd/carbon catalysts in 2-propanol. (**d**) Conversion and yield of FAL hydrogenation using Pd/CMK-5 in water.

To examine the effect of carbon supports on the Pd-catalyzed FAL hydrogenation, various carbon scaffolds, including OMCs (CMK-3, CMK-5, CMK-8, and MSU-F-C), multiwalled carbon nanotubes (CNTs), and commercial carbon (Vulcan SC), were prepared and evaluated (Figure 5) [25]. The structural order of the OMCs was confirmed using low-angle XRD (Figure 5a). CMK-3 and CMK-5 exhibited hexagonal *p6mm* structures similar to that of SBA-15, whereas CMK-8 displayed a cubic *Ia3d* structure corresponding to KIT-6. In contrast, MSU-F-C exhibits an uncharacteristic XRD pattern. Brunauer–Emmett–Teller (BET) surface area analysis revealed that the OMCs had significantly larger surface areas than CNT and Vulcan carbons. Pd catalysts were synthesized via chemical reduction using sodium borohydride in the presence of trisodium citrate to anchor small Pd clusters onto carbon supports. The TEM images confirm the successful synthesis of reverse replicas and the uniform dispersion of Pd NPs within the carbon support (Figure 5b). X-ray photoelectron spectroscopy (XPS) analysis revealed that metallic Pd^0^ species were predominant in the Pd NPs, which was a key factor in the catalytic activity.

Liquid-phase FAL hydrogenation was conducted using Pd catalysts and 2-propanol as the solvent. The Pd catalysts supported on OMCs exhibited higher FAL conversion rates than those supported on CNTs and Vulcan carbon (Figure 5c). Among the OMC-supported catalysts, Pd/CMK-5 exhibited the highest FAL conversion (100%), although the product selectivity remained similar across the OMCs. The superior performance of the Pd/OMCs can be attributed to their well-ordered pore structures and high surface areas, which facilitate faster mass transfer and enhanced absorption/diffusion processes. Notably, the two-dimensional hexagonal mesopore channels (*p6mm*) in Pd/CMK-3 and Pd/CMK-5 enabled higher mass-transfer rates owing to simpler internal pathways, whereas the interconnected cubic pores (*Ia3d*) in Pd/CMK-8 limited the mass transfer. The hexagonal hollow tubular framework of Pd/CMK-5 was particularly effective for FAL conversion. On the other hand, CPO emerged as the main product when water was used as the solvent, demonstrating that the solvent choice influenced product selectivity more than the carbon support structure (Figure 5d).

### 3.3. Interfacial Effect of Pd Supported on Mesoporous Oxides

Metal supported on oxide catalysts are the cornerstones of many essential catalysts used in chemical industries as well as carbon-supported catalysts [58,59,60]. Oxide supports play a critical role in stabilizing metal particles and influencing catalytic properties by enhancing stability, and providing adsorption sites for intermediates. However, the small surface area of metal oxides makes it challenging to disperse noble metals.

**Figure 6:**
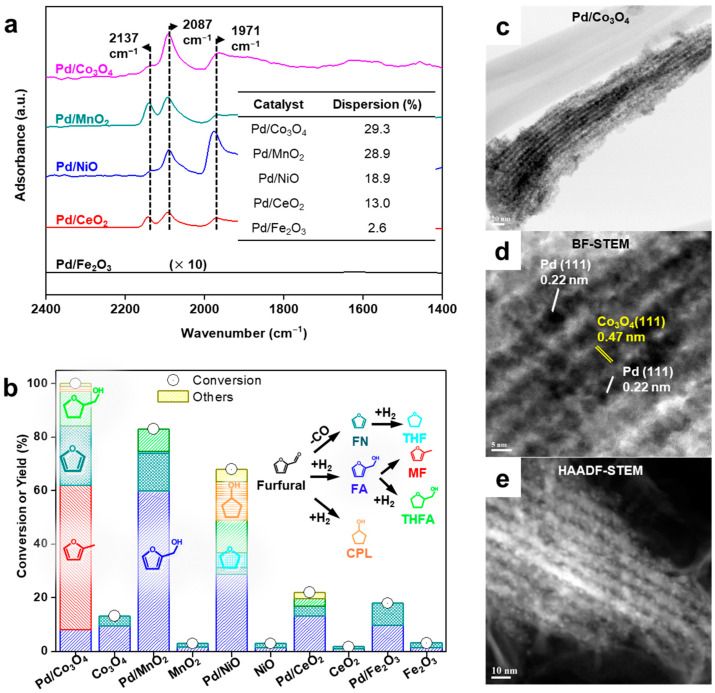
(**a**) CO adsorption DRIFT over supported Pd catalysts (inset; Pd dispersion over the Pd catalysts). (**b**) Hydrogenation of FAL over Pd/various metal oxide catalysts. (**c**–**e**) HAADF-STEM image of Pd/Co_3_O_4_.

**Figure 7:**
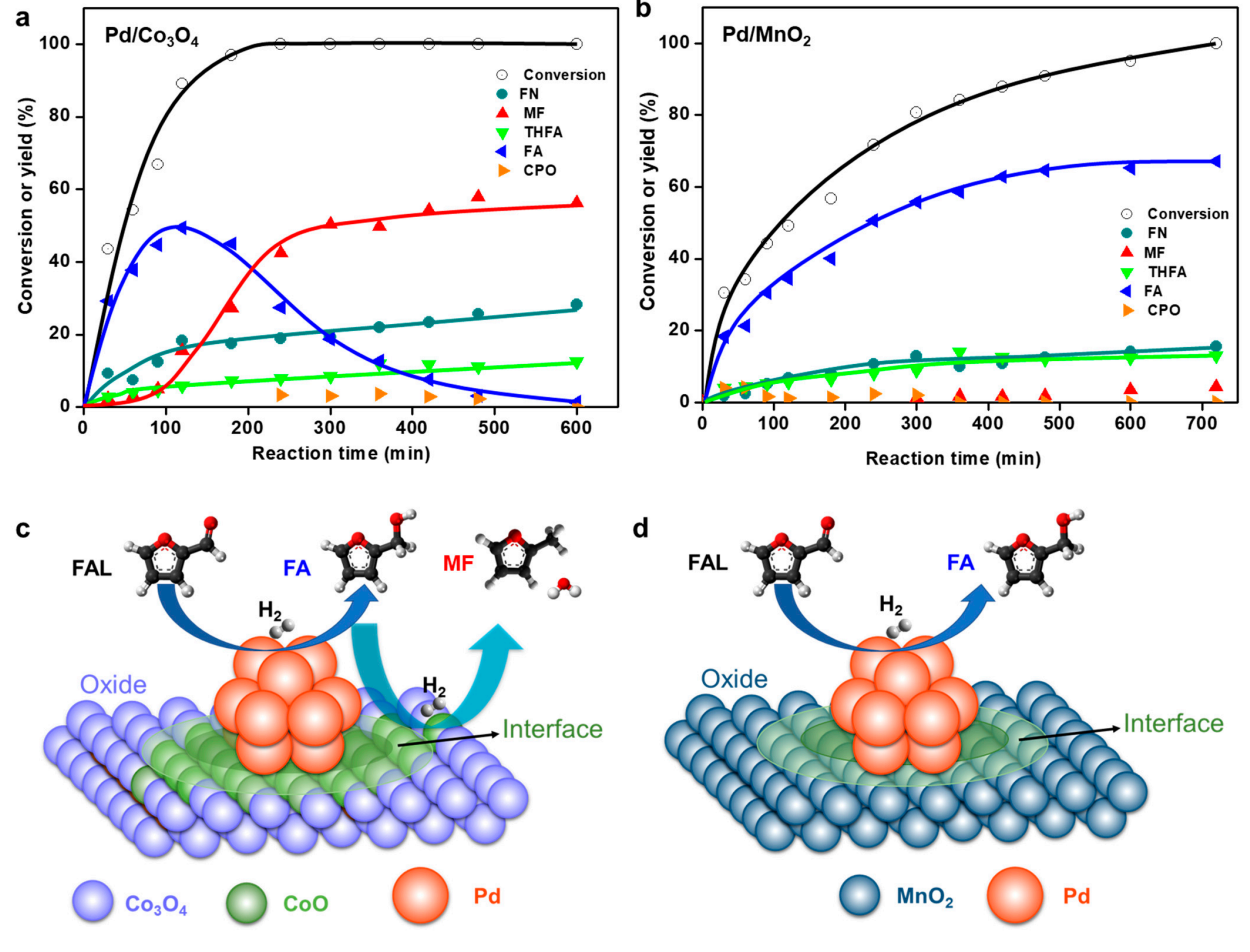
Product distribution of FAL as a function of reaction time over (**a**) Pd/Co_3_O_4_ and (**b**) Pd/MnO_2_. Illustration showing potential reaction sites and reaction pathways over (**c**) Pd/Co_3_O_4_ and (**d**) Pd/MnO_2_ catalysts during FAL hydrogenation.

To address this, mesoporous oxides such as Co_3_O_4_, MnO_2_, NiO, CeO_2_, and Fe_2_O_3_ were synthesized by nanocasting with mesoporous silica (SBA-15) as a template (Figure 6 and Figure 7) [26]. These oxides were impregnated with Pd NPs via chemical reduction to study their effects on the liquid-phase FAL hydrogenation. Inductively coupled plasma–optical emission spectroscopy (ICP–OES) confirmed successful loading of 5 wt% Pd onto the supports. CO temperature-programmed desorption (TPD) and CO adsorption diffuse reflectance infrared Fourier transform (DRIFT) spectroscopy revealed that Pd dispersion was approximately 30% for Co_3_O_4_ and MnO_2_, whereas it was less than 20% for the other supports (Figure 6a). Since the same silica template was used, the pore structures of the supports are expected to be similar. Therefore, this indicates that the nature of the oxide support significantly influenced the interaction with Pd, which in turn affected Pd dispersion. This suggests that Pd/Co_3_O_4_ and Pd/MnO_2_ would exhibit superior FAL conversions owing to their higher Pd dispersion. The TEM images confirmed the preservation of the mesoporous structures after Pd loading (Figure 6c–e), whereas the XRD patterns indicated that the original crystal structures of the oxides remained intact. A small peak corresponding to metallic Pd species was observed for Pd supported on CeO_2_ and Fe_2_O_3_. The pure oxide supports exhibited minimal FAL conversion (13% and 3% for Co_3_O_4_ and MnO_2_, respectively), whereas the Pd catalysts displayed significantly improved catalytic performance, with conversion rates in the order Pd/Co_3_O_4_ > Pd/MnO_2_ > Pd/NiO > Pd/CeO_2_ > Pd/Fe_2_O_3_ (Figure 6b). To further investigate the effect of porosity on catalytic performance, Pd catalysts supported on non-porous commercial oxides (Co_3_O_4_ and MnO_2_) were also prepared. These catalysts exhibited significantly lower Pd dispersion, leading to reduced FAL hydrogenation activity compared to their mesoporous counterparts. Interestingly, despite similar Pd dispersion, product selectivity was influenced by the type of oxide support. Pd/Co_3_O_4_ primarily produced MF, whereas Pd/MnO_2_ favored FA as the major product (Figure 7a,b). FA was used as the reactant to elucidate the reaction mechanism. Pd/Co_3_O_4_ readily converted FA to MF, whereas Pd/MnO_2_ was inactive in this conversion, suggesting that the metal–support interactions and redox properties of the oxide support play crucial roles in determining the reaction pathways and product distributions (Figure 7c,d).

### 3.4. Overview of Noble Metal Catalysis

The catalytic performance of noble-metal-based catalysts in FAL hydrogenation is strongly influenced by preparation methods, support materials, and structural properties. Pd/C catalysts synthesized via chemical reduction demonstrated superior activity and selectivity toward THFA due to their high dispersion and small Pd particle size, which enhanced hydrogenation efficiency.

Mesoporous carbon supports, particularly CMK-5 with its hollow tubular framework, further improved catalytic performance. While the type of carbon support had a limited effect on product selectivity, solvent choice significantly influenced reaction outcomes, highlighting the importance of both support structure and reaction conditions.

Additionally, mesoporous oxide supports played a crucial role in stabilizing Pd particles and influencing catalytic behavior through metal–support interactions. Factors such as oxidation state, redox properties, and electronic effects between Pd and oxides determined catalytic efficiency and product distribution. Notably, Pd/Co_3_O_4_ and Pd/MnO_2_ exhibited superior dispersion and conversion rates, with distinct selectivity patterns. These findings underscore the potential of tuning support properties to optimize catalytic performance in FAL hydrogenation.

Beyond these studies, researchers are actively exploring strategies to minimize noble metal usage by developing catalysts with highly dispersed active sites. For example, Xu et al. synthesized a Pt single-atom catalyst using cobalt–aluminum layered double hydroxide, achieving over 99% selectivity toward FA [61]. Taylor et al. successfully deposited Pt atoms on Cu NPs supported on Al_2_O_3_ via galvanic replacement, obtaining over 90% FA selectivity [62]. Additionally, Yuan et al. constructed a hierarchical Pd catalyst (Pd/CeO_2_/SiO_2_) that facilitated FAL conversion to CPO with 93% efficiency and 84% selectivity [63]. Another study by Gao et al. combined Pd (0.025 wt%) with Cu (3 wt%) to create well-dispersed PdCu NPs on MCM-41, yielding 94.6% FA [64]. Xu et al. synthesized a catalyst by mixing Pd, Pt, Ru, Mo, and Zn on TiO_2_ with total metal loading of 0.33 wt%, which showed higher FAL conversion and FA selectivity [65].

These advancements highlight that optimizing metal dispersion and support interactions can significantly enhance catalytic efficiency while reducing noble metal consumption. Moreover, the growing interest in transition-metal-based catalysts as potential alternatives to noble metal catalysts further underscores the importance of sustainable and cost-effective approaches in catalytic hydrogenation, a topic that will be explored in the following section.

## 4. Transition-Metal-Based Catalysts for FAL Hydrogenation

The development of catalysts for FAL hydrogenation that reduce reliance on noble metals and gaseous hydrogen is a critical challenge for commercialization [27,30]. Transition metal catalysts, particularly those based on Co, Cu, and Ni, have been extensively studied for FAL hydrogenation due to their tunable activity and selectivity. In this section, we categorize these catalysts into three main groups based on their composition and synthesis strategy. Monometallic catalysts (Section 4.1, Section 4.2 and Section 4.3) focus on individual transition metals, highlighting their unique catalytic properties and reaction mechanism [27,28,29]. Bimetallic catalysts (Section 4.4 and Section 4.5) explore synergistic effect between two metals such as Cu-Co and Cu-Ni, which enhance catalytic performance [30,31]. Lastly, MOF-based catalysts (Section 4.6 and Section 4.7) include both pristine MOFs and thermally treated MOF-derived materials, both of which offer high surface area and well-defined active sites, leading to improved hydrogenation efficiency [32,33]. This classification provides a comprehensive understanding of how different catalyst systems influence FAL conversion and selectivity.

### 4.1. Structure-Dependent Catalytic Properties of Mesoporous Cobalt Oxides

Among transition metal catalysts, mesoporous cobalt oxides garnered increasing attention due to their well-ordered pore structures and large surface areas, which enhance catalytic efficiency [66,67]. These materials not only provide high accessibility to active sites but also enable precise tuning of electronic properties via controlled reduction processes. The reduction of cobalt oxides, in particular, generates species with varied oxidation states, significantly affecting catalytic conversion and product selectivity [68,69]. The modulation of oxidation states is pivotal in enhancing their catalytic activity. In this study, we investigated how mesoporosity and cobalt oxidation states influence FAL hydrogenation performance.

**Figure 8:**
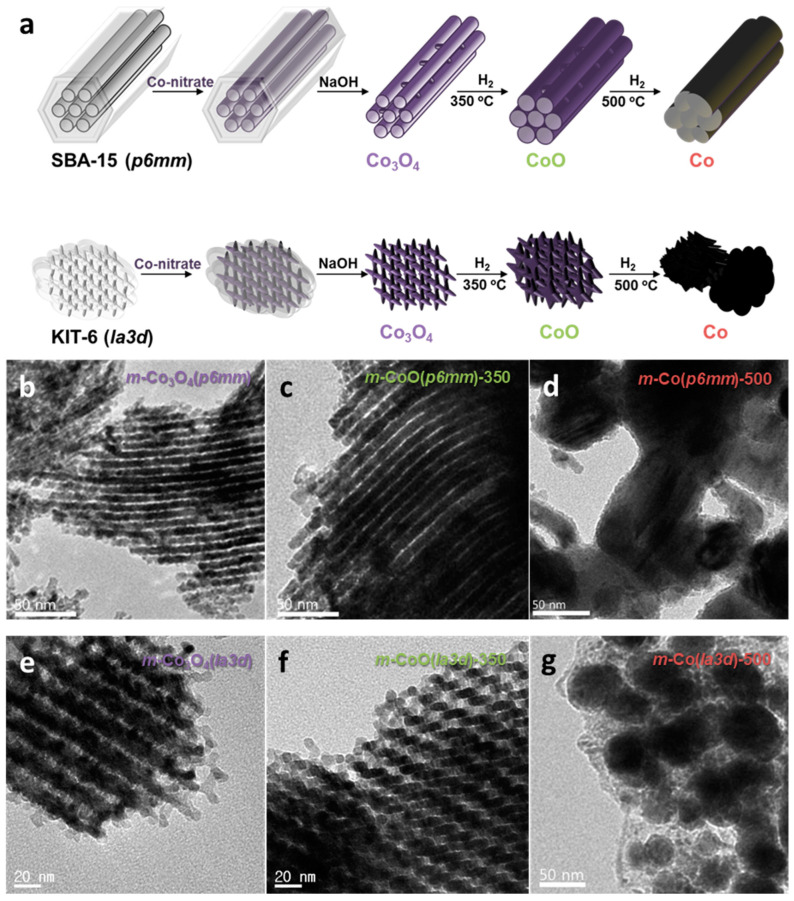
(**a**) Illustration of the nanocasting approach for the preparation of mesoporous Co_3_O_4_ (*m*-Co_3_O_4_) and its reduced structures (*m*-CoO and *m*-Co). TEM images of (**b**–**d**) *m*-Co_3_O_4_ (*p6mm*) and (e–g) *m*-Co_3_O_4_ (*Ia3d*) series prepared at different reduction temperatures: (**b**,**e**) without reduction treatment and with reduction at (**c**,**f**) 350 °C and (**d**,**g**) 500 °C.

**Figure 9:**
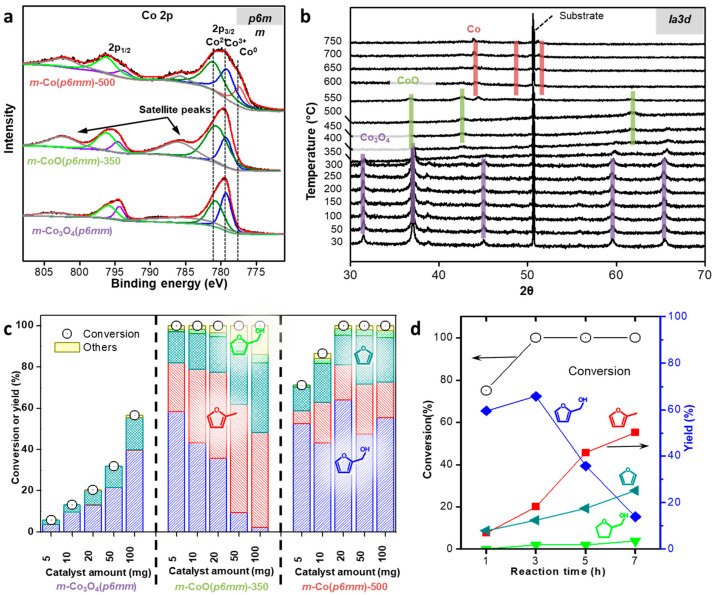
(**a**) Co 2*p* XPS spectra and (**b**) in situ XRD patterns of *m*-Co_3_O_4_ (*p6mm*). (**c**) Function of the reduction temperature and the amount of catalyst over *m*-Co_3_O_4_ (*p6mm*) series (5–100 mg). (**d**) Product distribution of FAL as a function of reaction time over *m*-Co_3_O_4_ (*p6mm*).

Mesoporous cobalt oxide (*m*-Co_3_O_4_) catalysts with controlled pore structures and crystallinities were synthesized using KIT-6 (*p6mm*) and SBA-15 (*Ia3d*) templates (Figure 8 and Figure 9) [27]. Reduction at 350 °C and 500 °C yielded *m*-CoO and *m*-Co, respectively, enabling investigation of structural and electronic effects (Figure 8a). Low-angle XRD and N_2_ physisorption confirmed the mesoporosity of these materials, although the TEM images revealed a partial collapse of the pore structures, resulting in reduced surface areas and pore volumes (Figure 8b–g). XPS provided insights into the chemical environment, with *m*-CoO showing the highest Co^2+^/Co^3+^ ratio among the catalysts studied. This ratio was notably higher for *m*-CoO (*p6mm*) at 2.33 compared to *m*-CoO (*Ia3d*) at 1.14, indicating the greater structural stability of the CoO crystal phase in the *p6mm* symmetry (Figure 9a). Furthermore, in situ XRD demonstrated the stability of the CoO phase up to 700 °C for *m*-CoO (*p6mm*) (Figure 9b). The catalytic performance was assessed in FAL hydrogenation (Figure 9c). While the spinel-phase Co_3_O_4_ catalysts converted only 13–16% of FAL, the reduced catalysts showed significantly higher activity. Among them, *m*-CoO outperformed both *m*-Co_3_O_4_ and *m*-Co, with *m*-CoO derived from the SBA-15 templates exhibiting superior performance to that derived from KIT-6. These findings highlight the critical role of reducibility, which varies with the pore structure and directly affects catalytic performance. The reaction mechanism was further examined using FA as the reactant. The results confirmed that CoO predominantly converted FA to MF and followed the reaction pathway to generate FR (Figure 9d). The selective hydrogenation of FAL to FA, followed by hydrogenolysis to MF, underscores the catalytic potential of CoO.

### 4.2. Cu_2_O (100) Surface as an Active Site

While Co-based catalysts facilitate hydrogenation and hydrogenolysis, Cu-based catalysts are particularly effective for FAL hydrogenation, often leading to the formation of FA as the main product with minimal byproducts [70,71]. The nature of active sites in Cu-catalyzed reactions remains debated because the phase and oxidation state of copper are highly influenced by the manufacturing method and reduction pretreatment conditions. Variations in the relative amounts of the Cu^0^, Cu^+^, and Cu^2+^ species make it challenging to identify the active phase of Cu-based catalysts.

**Figure 10:**
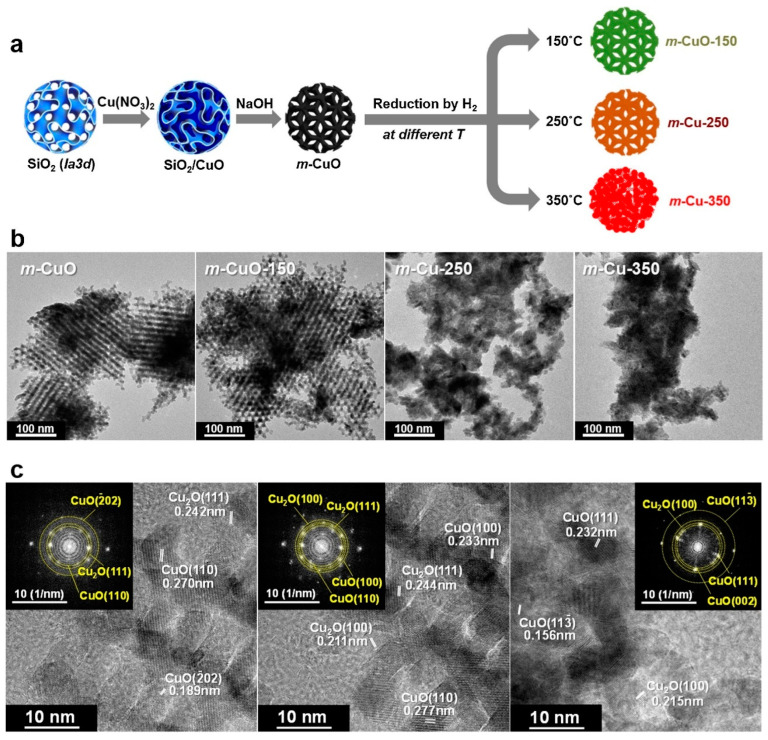
(**a**) Illustration of the preparation of *m*-CuO and its reduced structures. (**b**) TEM images of *m*-CuO series prepared at different reduction temperatures: as-prepared *m*-CuO, *m*-CuO-150, *m*-Cu-250, and *m*-Cu-350. (**c**) HR-TEM images of *m*-CuO-150 with the corresponding FFT patterns.

**Figure 11:**
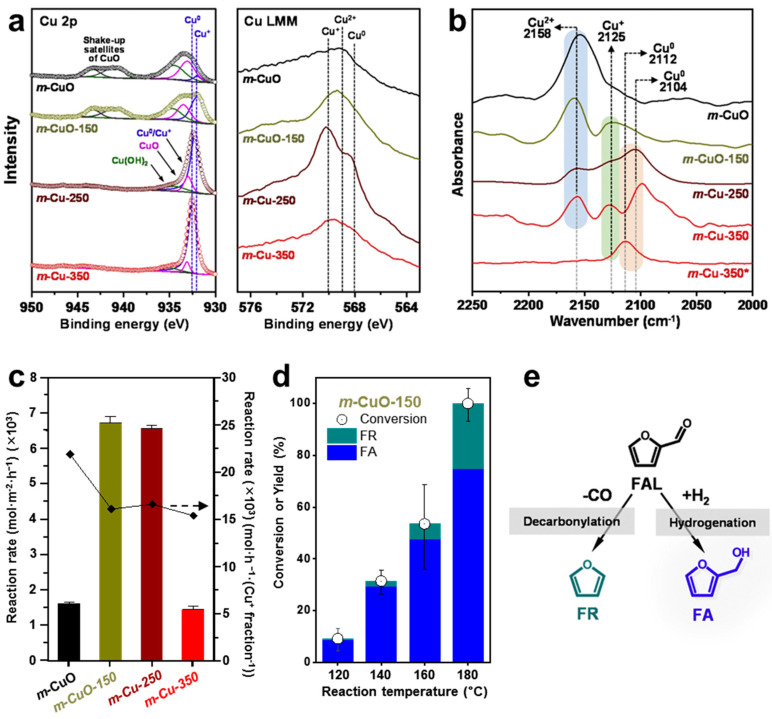
(**a**) Cu 2*p*_3/2_ XPS and Cu LMM Auger peaks for *m*-CuO series. (**b**) CO adsorption DRIFT spectra of *m*-CuO series (* in situ reduced to avoid re-oxidation before analysis). (**c**) Surface activity and reaction rate for FAL hydrogenation over *m*-CuO series. (**d**) Product distribution of FAL as a function of reaction temperature over *m*-CuO-150. (**e**) Illustration showing potential reaction pathways over *m*-CuO series.

Therefore, in this study, mesoporous metal oxide catalysts with controlled pore structures and oxidation states were synthesized to investigate the role of the active sites in Cu-catalyzed FAL hydrogenation (Figure 10 and Figure 11) [28]. A replica of CuO with a cubic pore structure (*Ia3d*) was synthesized and subjected to controlled reduction by hydrogen, sequentially converting mesoporous CuO (*m*-CuO) to *m*-Cu_2_O, and ultimately to *m*-Cu (Figure 10a). Although numerous studies have investigated Cu-based catalysts to identify active surface sites, the highly dispersed Cu species on the support make it difficult to accurately characterize these sites because of their small proportion compared to the bulk support. Mesoporous CuO, with its large surface area and well-defined pore structure, can form distinct surface species, including Cu^0^, Cu^+^, and Cu^2+^, under reducing conditions. In this study, mesoporous copper oxide was synthesized from KIT-6 and reduced at three different temperatures (150, 250, and 350 °C). The TEM images confirmed the well-structured porous nature of the materials, although phase transformation and partial collapse of the structure occurred as the reduction temperature increased (Figure 10b). The FFT pattern obtained from the high resolution-TEM of *m*-CuO-150 revealed the presence of a Cu_2_O phase mixed with CuO (Figure 10c), which was also confirmed by XRD analysis. The surface electronic structure was examined using XPS Cu 2*p* and Auger Cu LMM spectra (Figure 11a). With an increase in the reduction temperature, the proportions of Cu^+^ and Cu^0^ increased, while that of Cu^2+^ decreased. The CO adsorption DRIFT spectra revealed that *m*-CuO-150 contained the highest proportion of Cu^+^ species (42.8%), followed by *m*-Cu-250 (41.0%) (Figure 11b).

Catalytic FAL activation was shown to correlate with Cu_2_O content, as the surface activity for FAL activation increased with the presence of Cu_2_O (Figure 11c). The reaction temperature also influenced conversion, with higher temperatures leading to higher conversion rates (Figure 11d). However, excessively high temperatures promoted the decarbonylation of FAL to FR, which resulted in lower selectivity for FA (Figure 11e). These findings underline the importance of mesoporous metal oxides with controlled electronic states in metal components.

### 4.3. Ni-Doped Ordered Mesoporous Carbon Catalysts

While electronic state modulation significantly influences catalytic performance, long-term stability is equally critical, particularly in supported transition-metal-based systems. One of the main challenges in maintaining catalytic stability is the agglomeration of metal NPs, which leads to a loss of active surface area and reduced performance over time [72]. This phenomenon is commonly observed in supported Ni-based catalysts, which are highly effective for FAL hydrogenation due to their strong H_2_ activation ability [35]. However, without proper structural control, Ni NPs tend to aggregate, diminishing their catalytic efficiency. To address this issue, carbon-based supports have been widely explored, as discussed in Section 3.2. In particular, OMC supports provide a high surface area and uniform pore distribution, effectively preventing Ni particle agglomeration. Furthermore, embedding Ni precursors directly into the OMC framework during synthesis can further enhance dispersion, leading to improved catalytic activity and stability.

**Figure 12:**
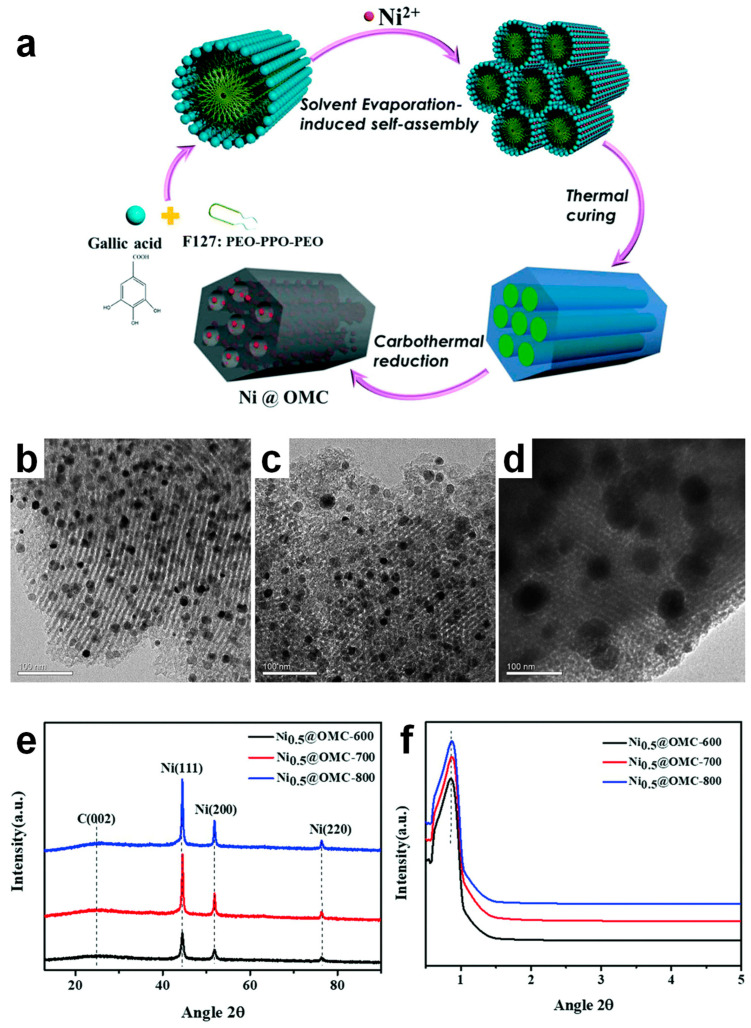
(**a**) Schematic illustration of synthesis procedure of Ni@OMC nanocomposites. TEM images of (**b**) Ni_0.5_@OMC-600, (**c**) Ni_0.5_@OMC-700, (**d**) Ni_0.5_@OMC-800. (**e**) Wide- and (**f**) small-angle XRD patterns of Ni@OMC obtained at different calcination temperatures.

**Figure 13:**
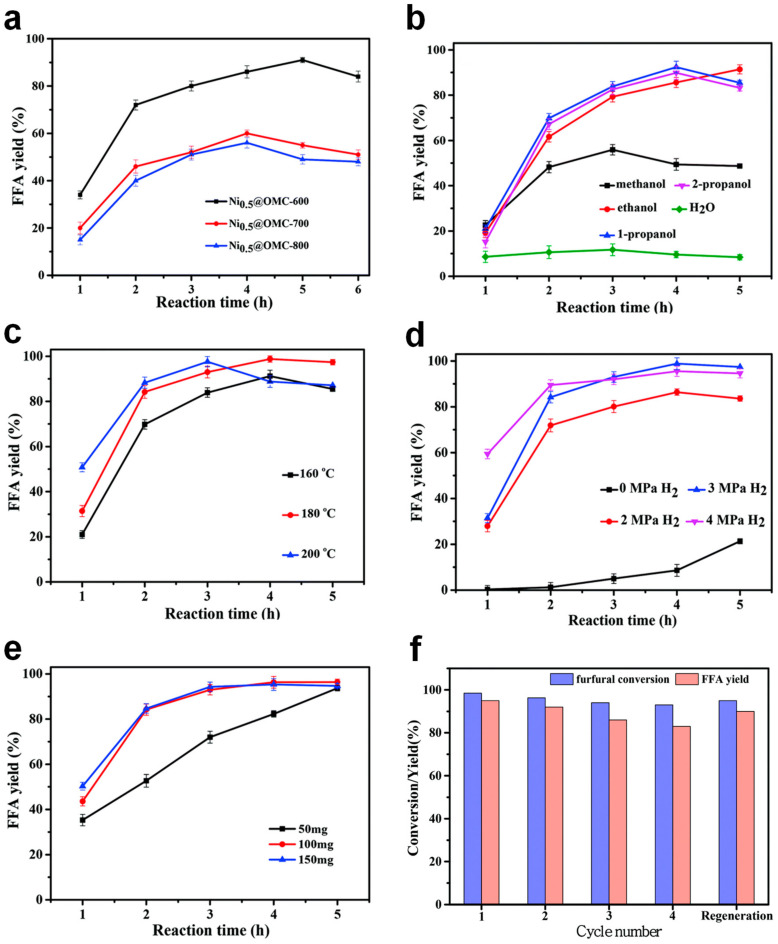
(**a**) Catalytic FAL hydrogenation to FA as a function of time over Ni_0.5_@OMCs. Effects of (**b**) solvent, (**c**) temperature, (**d**) hydrogen pressure, and (**e**) catalyst loading in FAL hydrogenation. (**f**) Catalyst recycling tests.

Building on this concept, Tang et al. synthesized agglomeration-suppressed Ni embedded OMC (Ni@OMC) catalysts by one pot EISA method where gallic acid, Ni^2+^ ions, and F127 were used as carbon precursor, crosslinker, and soft template, respectively, followed by carbonization and reduction (Figure 12 and Figure 13) [29]. The amount of Ni salt varied from 0.1 to 1.0 g, and a mesoporous structure was observed only when 0.5 g of Ni salt were used. This suggests that the ratio of Ni^2+^ to crosslinker played a pivotal role in the mesopore formation. The impact of carbonization temperature (600, 700, and 800 °C) was also investigated (Figure 12b–d). While the mesoporous structure remained intact, Ni particle size increased with rising temperature, as confirmed by wide-angle XRD (Figure 12e). Additionally, small-angle XRD revealed a well-ordered 2D hexagonal *p6mm* mesoporous structure (Figure 12f), while N_2_ physisorption analysis demonstrated a uniform pore distribution in Ni@OMCs.

Catalytic FAL hydrogenation was assessed using Ni_0.5_@OMC catalysts carbonized at different temperatures. Ni_0.5_@OMC-600 exhibited the highest FAL conversion and FA selectivity over the investigated reaction period (Figure 13a). The influence of solvent polarity was also examined by testing H_2_O and C_1_–C_3_ alcohols. Interestingly, FAL conversion increased with solvent polarity. However, FA selectivity was the highest when 1-propanol was used as a solvent, whereas H_2_O and methanol caused undesired byproducts by participating in the reaction (Figure 13b). The effects of reaction parameters, including temperature, hydrogen pressure, and substrate-to-catalyst ratio, were also explored (Figure 13c–e). Higher reaction temperature and pressure enhanced FAL conversion. However, FA selectivity exhibited an optimum at intermediate conditions, indicating that excessive temperature and pressure favored side reactions. Increasing the catalyst amount improved both FAL conversion and FA selectivity, but beyond a certain threshold, further enhancement was not observed. Reusability tests demonstrated that the catalyst maintained its mesoporous structure after four cycles, with only slight Ni agglomeration observed, which might contribute to a slight decrease in FAL conversion and FA selectivity (Figure 13f).

### 4.4. Mesoporous Mixed CuCo Oxides as Robust Catalysts

While monometallic catalysts such as Co-, Cu-, and Ni-based systems have demonstrated promising activity in FAL hydrogenation as shown in Section 4.1 through Section 4.3, they often suffer from limitations, particularly in terms of stability under pretreatment conditions. For example, mesoporous metal oxides, despite their high surface area and tunable properties, can undergo structural degradation in hydrogen-rich environments [73]. To overcome these challenges, bimetallic catalysts have emerged as robust alternatives, offering enhanced stability and synergistic effects that monometallic systems lack [74,75]. Among them, mixed metal oxides such as Cu-Co catalysts have shown exceptional performance in FAL hydrogenation [76,77]. These systems not only strengthen the structural integrity of the catalyst but also introduce new active species, enabling unique reaction pathways. In particular, the strong interaction between copper and cobalt promotes the cleavage of C–O bonds, a critical step in hydrogenation reactions, making Cu-Co catalysts highly effective for this transformation.

**Figure 14:**
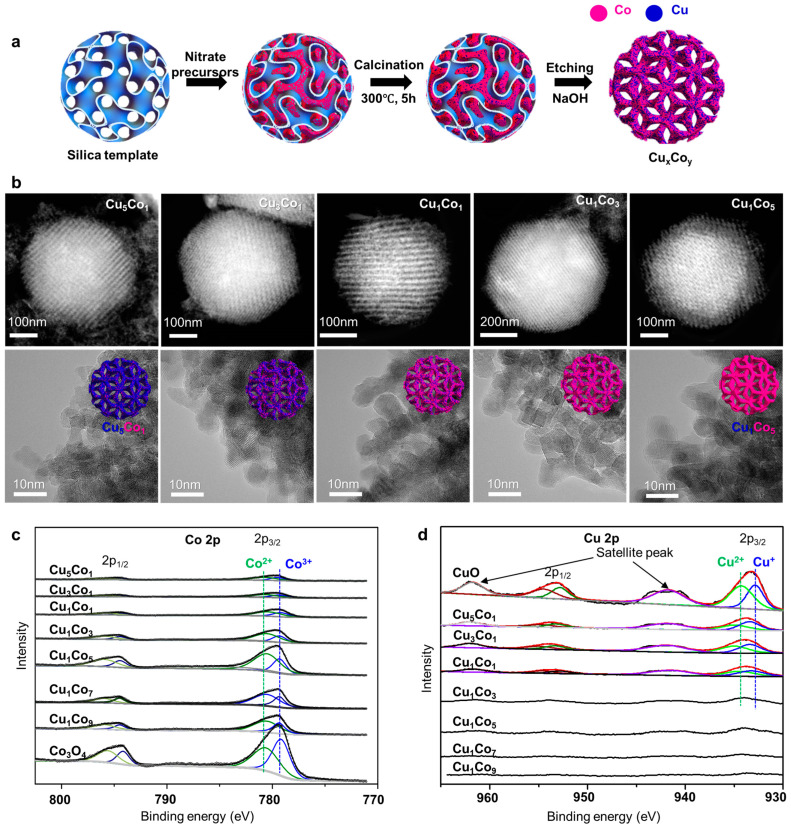
(**a**) Schematic illustration of the preparation of mesoporous mixed CuCo oxides: Co in pink and Cu in blue. (**b**) HAADF-STEM and HR-TEM images of mesoporous Cu_x_Co_y_ oxides. (**c**) Co 2*p* and (**d**) Cu 2*p* XPS spectra.

To synthesize mesoporous Cu-Co mixed oxides, cubic *Ia3d* mesoporous silica (KIT-6) was employed as a template using the nanocasting method (Figure 14a) [30]. TEM and high-angle annular dark field scanning TEM (HAADF-STEM) images confirmed the successful casting of mesoporous Cu_x_Co_y_ mixed oxides (Figure 14b), with energy dispersive spectroscopy (EDS) mapping demonstrating the uniform mixing of Cu and Co species without noticeable phase segregation. Mesoporosity was further confirmed by N_2_ physisorption, which showed a characteristic hysteresis loop typical of mesoporous materials. XPS revealed that Cu was present in the 1^+^ and 2^+^ oxidation states, whereas Co was present in the 2^+^ and 3^+^ states (Figure 14c,d). XRD analysis showed that the patterns of the mixed oxides deviated from those of the pure oxides (CuO and Co_3_O_4_), suggesting the mutual doping of Cu and Co ions, with no metallic phases observed.

**Figure 15:**
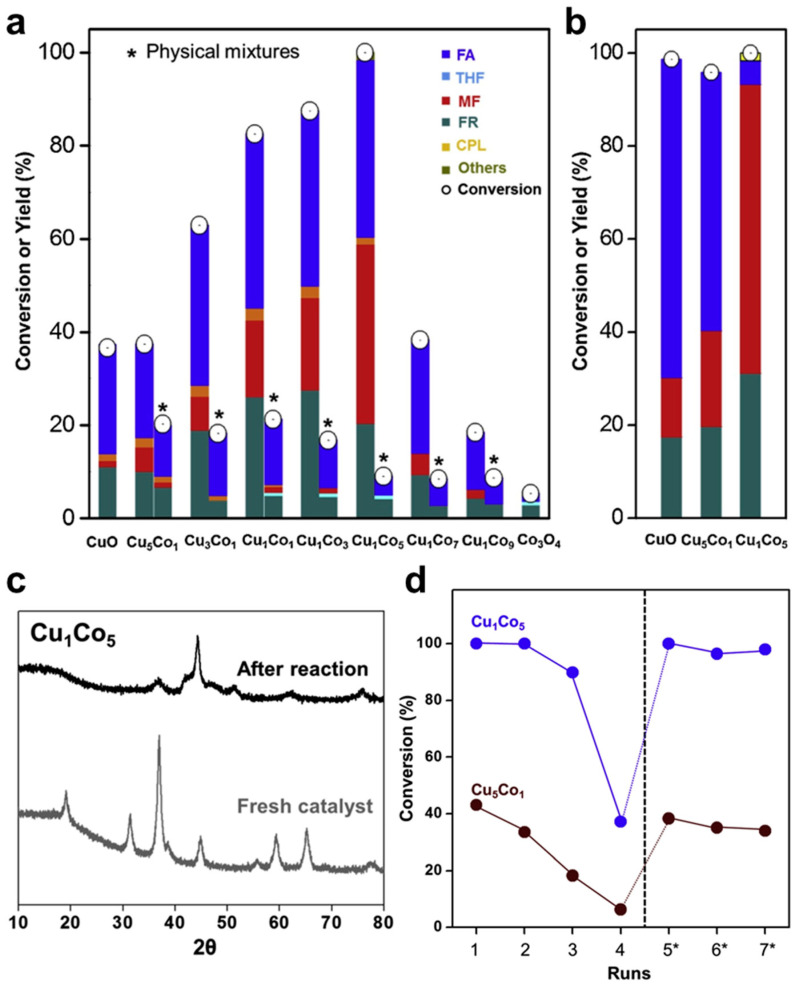
(**a**) FAL conversion and yield as a function of reaction time over mesoporous Cu_x_Co_y_ catalysts. (**b**) FAL conversion and yield after the 10 h reaction over CuO, Cu_5_Co_1_, and Cu_1_Co_5_ catalysts. (**c**) XRD patterns of fresh and spent Cu_1_Co_5_ catalyst. (**d**) Recycling test over Cu_1_Co_5_ and Cu_5_Co_1_ catalysts (* reactivated by calcination at 300 °C for 2 h).

The catalytic performance of the mesoporous Cu-Co mixed oxides in FAL hydrogenation was evaluated and compared with that of single metal oxides (CuO and Co_3_O_4_) and their physical mixtures in varying proportions (Figure 15a). Volcano curves revealed an optimal Cu/Co ratio, with Cu_1_Co_5_ exhibiting the highest FAL conversion. Interestingly, the physical mixtures showed much lower activities than the mixed oxides, highlighting the crucial role of Cu-Co interaction in enhancing catalytic performance. Moreover, as the Co content increased up to Cu_1_Co_5_, MF selectivity also increased, demonstrating metal-dependent product selectivity (Figure 15b). Unlike monometallic oxides, the Cu-Co mixed oxides were used without pre-reduction treatment and underwent in situ reduction during the reaction, as confirmed by XRD patterns of the spent catalysts (Figure 15c). This enhanced metal–metal interaction facilitated a spontaneous phase transition, further contributing to the superior catalytic performance of Cu-Co catalysts. Reusability tests showed that catalyst reactivation by calcination under air effectively maintained stable FAL conversion over four cycles (Figure 15d).

### 4.5. Role of Cu^+^ and CuNi Alloy in Mesoporous CuNi Catalysts

The strong synergistic effects observed in Cu-Co mixed oxides highlight the potential of bimetallic system in enhancing catalytic performance. Similarly, bimetallic CuNi catalysts have demonstrated advantages over their monometallic counterparts [78,79]. As described in Section 4.2 and Section 4.3, Cu_2_O phase and reduced Ni species serve as active sites for FAL hydrogenation. However, when Cu and Ni oxides are mixed, additional factors come into play, such as the formation of CuNi alloy and the role of Cu^+^ species. These aspects require further investigation, as mixed metal oxides exhibit distinct properties compared to physical mixtures of their respective oxides, as seen in the Cu-Co system.

**Figure 16:**
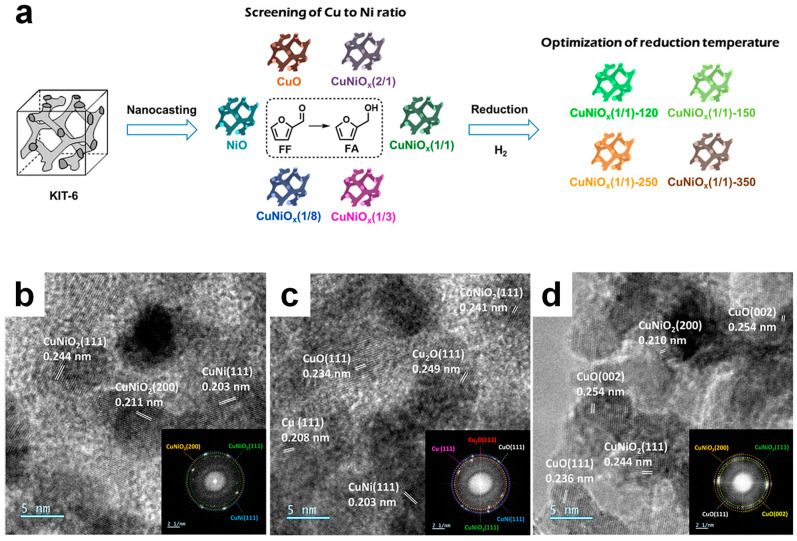
(**a**) Schematic illustration of the synthesis and screening approach of active mesoporous CuNiO_x_ for FAL hydrogenation. (**b**–**d**) TEM images of CuNiO_x_(1/1)-150 with the corresponding FFT patterns.

**Figure 17:**
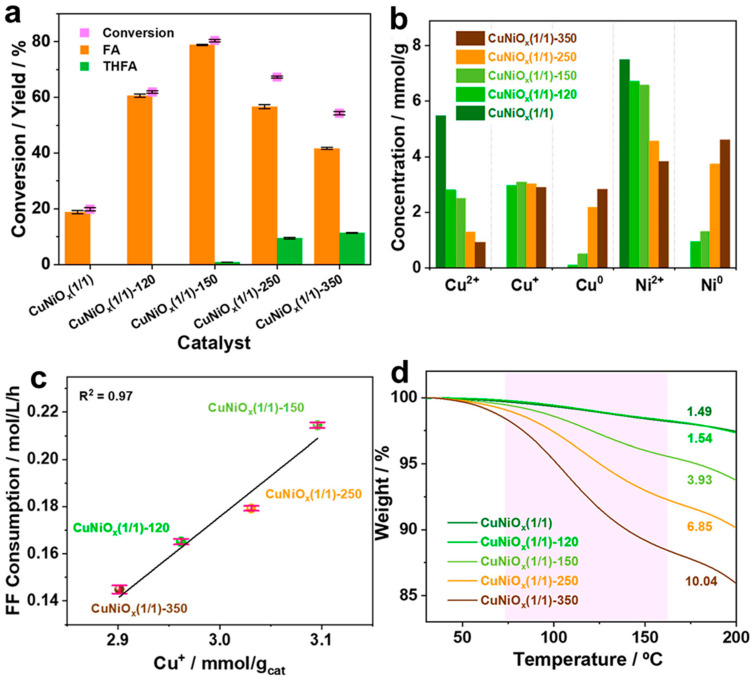
(**a**) Catalytic performance of as-synthesized and reduced CuNiO_x_(1/1) catalysts in FAL hydrogenation. (**b**) Concentration of Cu and Ni with different oxidation states in the as-synthesized and reduced CuNiO_x_(1/1) catalysts. (**c**) Correlation of FAL consumption and Cu^+^ concentration. (**d**) Thermogravimetric profiles of FAL-adsorbed catalysts, where pink range represents FAL desorption area.

**Figure 18:**
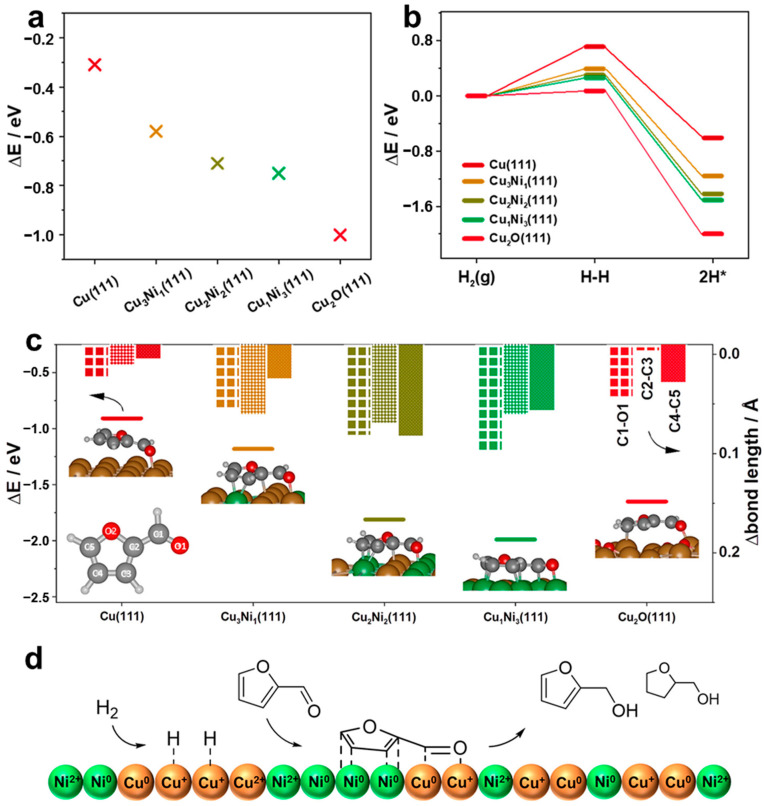
(**a**) Calculated adsorption energies of the H atom. (**b**) Dissociation energetics of gas phase H_2_ on different Cu and Ni species. (**c**) Comparison of increased bond length of FAL adsorbed on different Cu and Ni species. (**d**) Scheme of a possible FAL hydrogenation mechanism.

In this study, mesoporous Cu-Ni mixed oxides with varying Cu/Ni ratios were synthesized via the nanocasting method using KIT-6 as a template (Figure 16, Figure 17 and Figure 18) [31]. The catalysts were then reduced at different temperatures to create several phases. The as-synthesized CuNiO_x_ catalysts were characterized by XRD, N_2_ physisorption, and XPS, confirming their mesoporous structures and the presence of Cu^2+^ and Ni^2+^ ions. Initial screening of different Cu/Ni ratios for FAL hydrogenation proved that CuNiO_x_ with a 1:1 ratio exhibited the best performance while maintaining its pore structure (Figure 16). The catalyst with the optimal ratio was further reduced at four different temperatures and tested for FAL hydrogenation. The catalyst reduced at 150 °C displayed the highest FAL conversion and FA yield (Figure 17a). Direct reuse of the catalyst resulted in a slight loss of activity, while preserving its original morphology, as confirmed by TEM.

The quantitative analysis of Cu and Ni oxidation states revealed Cu^+^ species played a crucial role in FAL conversion. The CuNiO_x_ with a 1:1 ratio reduced at 150 °C contained the highest amount of Cu^+^ species (Figure 17b) and exhibited superior H_2_ adsorption and dissociation ability compared to other catalysts, suggesting that Cu^+^ sites served as the primary H_2_ activation sites, leading to the highest FAL conversion (Figure 17c). Additionally, calculations of hydrogen atom adsorption energy and H_2_ dissociation energy demonstrated that Cu_2_O phase effectively dissociates H_2_ and strongly adsorbs hydrogen atoms (Figure 18a,b). The adsorption behavior of FAL was further investigated by TGA and IR, proving that the catalyst reduced at higher temperatures displayed stronger FAL adsorption (Figure 17d). DFT calculations also indicated CuNi alloy phase was the main sites for FAL adsorption. Combining the experimental and computational results, it was demonstrated that both Cu^+^ and CuNi alloy phase were responsible for hydrogen and FAL activation, proposing a possible FAL hydrogenation mechanism (Figure 18d).

### 4.6. Role of Metal Coordination on MOF Catalysts

While bimetallic systems, such as Cu-Co and Cu-Ni catalysts, have demonstrated promising activity in FAL hydrogenation, alternative catalytic approaches have also been explored to improve catalytic efficiency. Among them, MOFs have emerged as a versatile platform for designing highly tunable catalytic systems [80,81,82]. In particular, Zr-based MOFs have been investigated for the hydrogenation of biomass-derived compounds, especially in catalytic transfer hydrogenation (CTH) [83,84]. CTH offers a potential alternative to conventional hydrogenation by utilizing organic molecules as a hydrogen source, which undergo oxidation to generate active hydrogen species. Although Zr-MOFs exhibit catalytic activity in CTH, their structural stability under high reaction temperatures remains a challenge that needs to be addressed.

**Figure 19:**
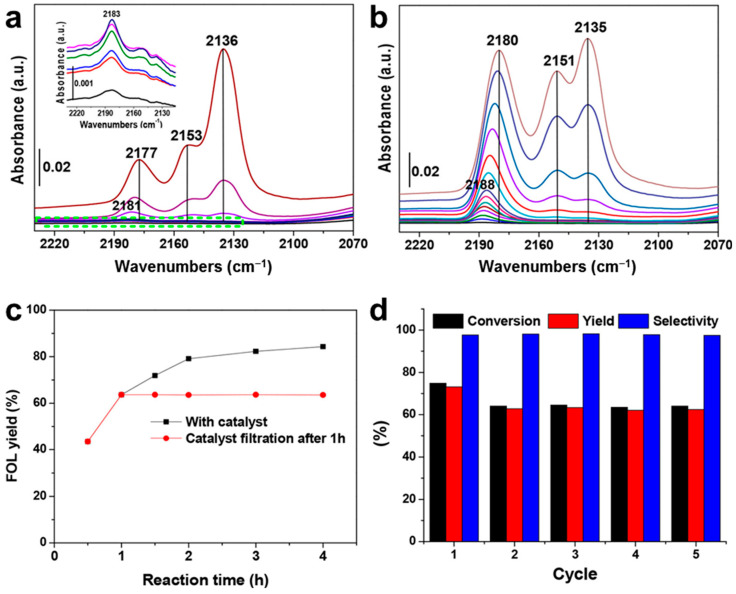
FT-IR spectra of (**a**) MOF-808 and (**b**) M-MOF-808 obtained at −173 °C during CO adsorption. Magnified FT-IR spectra of green dotted box (inset of (a)). (**c**) Hot filtration test and (**d**) catalyst recycling test using M-MOF-808.

To complement the stability of MOF-based catalysts, Valekar et al. explored the CTH of FAL using a series of Zr-MOF catalysts with different ligand coordinations to improve catalytic activity at low temperatures (Figure 19 and Table 1) [32]. The synthesized Zr-MOFs were highly crystalline and porous, with well-defined pore diameters. UiO-66, UiO-67, and DUT-52, which have a coordination number of 12, showed relatively low FAL conversion of approximately 2–5% at 82 °C (Table 1 Entry 1–3). However, decreasing the metal node connectivity led to a significant increase in FAL conversion, reaching 13.5% for DUT-67 (8-coordinated, Table 1 Entry 4) and 81.3% MOF-808 (6-coordinated, Table 1 Entry 5). This implies that ligand coordination at the metal node significantly influences the CTH of FAL.

MOF-808, which exhibited the highest FAL conversion, was further modified by methanol activation (M-MOF-808). Characterization revealed that M-MOF-808 possessed a greater number of CO adsorption sites than unmodified MOF-808, which likely facilitated the adsorption and conversion of FAL molecules (Figure 19a,b). As a result, M-MOF-808 achieved an impressive FAL conversion of 89.3% at 82 °C in 2 h (Table 1 Entry 7) and 96.5% at 40 °C in 24 h (Table 1 Entry 8), whereas unactivated MOF-808 exhibited only 27.5% conversion under the same low-temperature conditions (Table 1 Entry 6). The enhanced activity was attributed to the increased interaction between the carbonyl group and the active sites, which facilitated FAL conversion. Furthermore, the authors extended their investigation to the CTH of other carbonyl compounds using M-MOF-808, achieving >90% conversion in the hydrogenation of carbonyl groups to hydroxyl ones. Catalyst was filtered out after 1 h reaction, and FA yield remained unchanged, demonstrating heterogeneous characteristics of M-MOF-808 (Figure 19c). Furthermore, FAL conversion decreased in the second cycle but remained stable over subsequent three cycles, suggesting that some active sites were blocked during the initial reaction (Figure 19d).

### 4.7. Structural Evolution of ZIF-67-Derived Catalysts

The as-synthesized MOFs can be directly used as catalysts for catalytic reactions, as demonstrated in the previous section. However, thermal treatment of MOFs has emerged as an effective strategy to enhance catalytic performance by creating well-dispersed active sites. Among these, ZIF-67 has been widely explored as a precursor for Co-based catalysts [54,55]. Its well-defined structure provides a framework for generating highly dispersed Co catalysts supported on nitrogen-doped carbon (Co/NC). However, under high-temperature reduction conditions, the overall structure of ZIF-67 collapses, leading to potential loss of surface area and porosity. Therefore, achieving a uniform distribution of active Co species within the porous carbon network while preserving a large surface area is crucial for optimizing catalytic performance.

**Figure 20:**
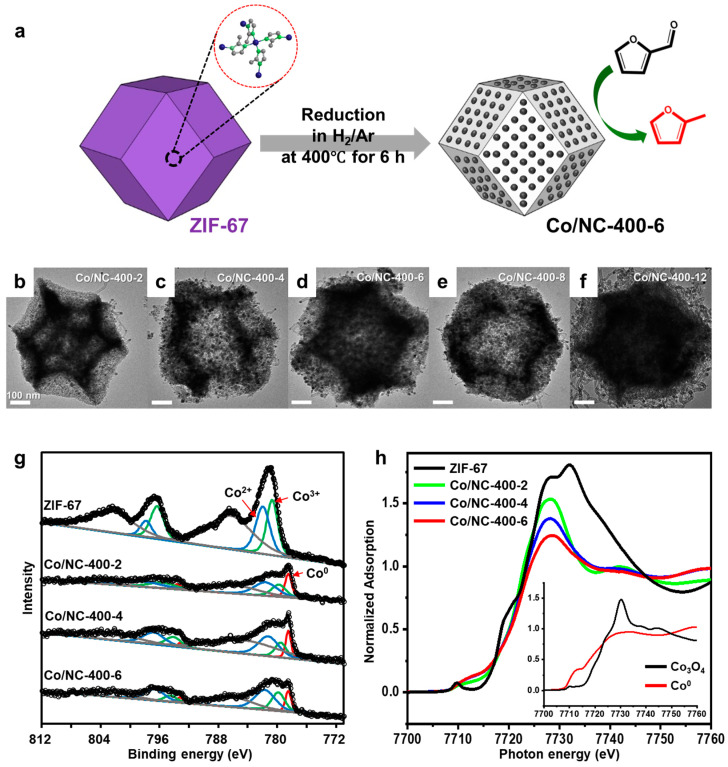
(**a**) Schematic illustrations of the transformation of ZIF-67 into a Co supported on a *N*-doped carbon catalyst (Co/NC) by H_2_/Ar reduction. (**b**–**f**) TEM images of ZIF-67 as a function of the H_2_ reduction time at 400 °C: (**b**) Co/NC-400-2, (**c**) Co/NC-400-4, (**d**) Co/NC-400-6, (**e**) Co/NC-400-8, and (**f**) Co/NC-400-12. (**g**) Co 2*p* XPS and (**h**) normalized Co K-edge XANES of ZIF-67 and Co/NC-400 series.

To address this challenge, various ZIF-67-derived Co/NC catalysts were prepared via H_2_ reduction under different temperatures and durations (Figure 20a) [33]. The structural evolution of the catalysts was analyzed using TEM. Catalysts reduced at temperatures above 400 °C exhibited structural changes distinct from the pristine ZIF-67. While the polyhedral structure of ZIF-67 was preserved after reduction at 400 °C for up to 8 h, severe shrinkage and carbon nanotube growth were observed after 12 h (Figure 20b–f). BET analysis revealed a significant decrease in surface area with prolonged reduction time. XPS and XANES spectra were obtained to determine the oxidation state of the Co species in the catalyst (Figure 20g,h). The longer the reduction duration, the greater the conversion of Co^2+^ to metallic Co, with pristine ZIF-67 exhibiting the most oxidized Co state.

**Figure 21:**
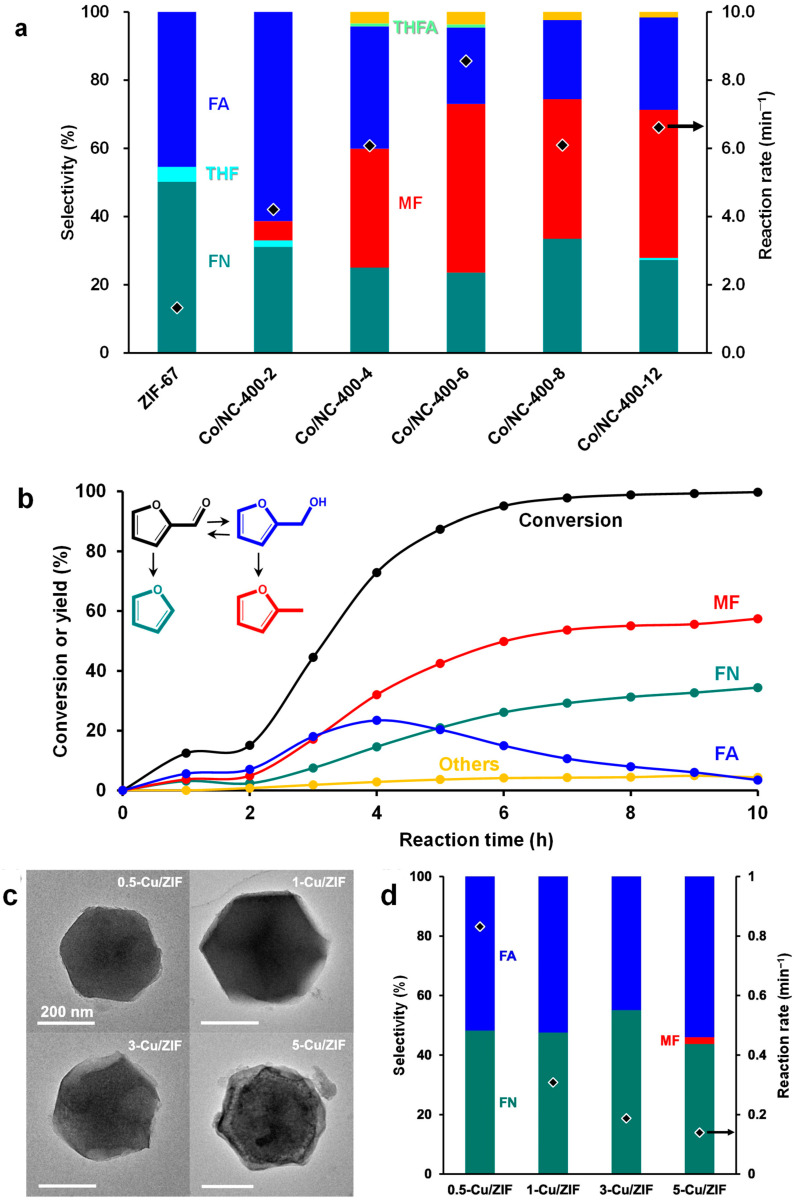
(**a**) Reaction rate and selectivity for FAL hydrogenation over ZIF-67 and Co/NC-400 series. (**b**) FAL conversion and selectivity as a function of the reaction time over Co/NC-400-6. (**c**) TEM images and (**d**) FAL hydrogenation performance of Cu-incorporated ZIF-67.

The catalytic performance of both the reduced Co/NC catalysts and pristine ZIF-67 were evaluated in the hydrogenation of FAL (Figure 21a). The highest FAL conversion was achieved using Co/NC-400-6, whereas ZIF-67 showed minimal FAL conversion. This suggests that Co^2+^, when strongly coordinated with organic linkers, is ineffective for activating FAL. A slight decrease in FAL conversion was observed over four cycles, likely due to the gradual increase in Co particle size, as confirmed by characterization of the spent catalysts. The time-course study using Co/NC-400-6 suggested two parallel reaction pathways: one leading to FR through the FA intermediate and another directly forming MF (Figure 21b). To further tune the catalytic selectivity, Cu, a well-known active component in FAL hydrogenation to FA, was incorporated into the ZIF-67 structure (Figure 21c,d). The incorporation of Cu significantly shifted product selectivity toward FA, demonstrating its role in controlling reaction pathways.

### 4.8. Overview on Transition Metal Catalysis

The effects of mesoporous structure and porosity on FAL hydrogenation using transition metal catalysts were systematically analyzed. The mesoporous CoO catalyst with *p6mm* symmetry exhibited a higher reaction rate than Co_3_O_4_ and Co, highlighting the significance of cobalt oxidation state, mesopore symmetry, and reduction conditions. The distribution of Cu species (Cu^0^, Cu^+^, and Cu^2+^) in mesoporous CuO catalysts was also investigated, revealing that a higher proportion of Cu^+^ species correlated with increased catalytic activity. Unlike traditional supported catalysts, the nanocasting method enabled precise characterization of active surface species. Additionally, Ni catalysts synthesized via EISA provided OMC structures, and reaction condition optimization led to high FA yield, emphasizing the importance of mesoporosity.

In addition, mesoporous mixed Cu-Co oxides were examined to understand active components and factors influencing efficiency and selectivity. Notably, unlike many transition metal catalysts, Cu-Co mixed oxides did not require pre-reduction treatment before FAL hydrogenation, demonstrating their intrinsic activity under reaction conditions. That is, the intimacy between Cu and Co species was one of the important factors. Similarly, CuNiO_x_ catalysts were investigated through detailed characterization and DFT calculations, showing that Cu^+^ species facilitated H_2_ activation, while CuNi alloy phase promoted FAL adsorption. The strong interplay between these two sites resulted in a synergistic effect. These findings underscore the importance of bimetallic interactions in tailoring catalytic performance.

Furthermore, both pristine and treated MOF-based catalysts were explored. Study on Zr-MOFs demonstrated that lower metal–linker connectivity improved reactant accessibility, leading to enhanced catalytic performance. In addition, Co/NC catalysts derived from ZIF-67 revealed that strong interactions between Co^2+^ and linker hindered FAL hydrogenation, while thermal treatment weakened these interactions, enabling efficient FAL conversion. These studies highlight that reactant accessibility, in line with the role of mesoporosity, is a key factor in optimizing catalytic performance.

In addition to the above studies, extensive research on transition metal catalysis has been conducted. For example, Kang et al. proposed a trimetallic NiZrCo catalyst derived from layered double hydroxide for the selective CTH of FAL to FA [85]. The optimized catalyst achieved a 76.6% FA yield within 2 h, with a slight loss after five cycles. Jia et al. prepared a series of Cu-M/Al_2_O_3_ (M=Fe, Co, Ni, or Ce) catalysts and investigated the role of the additive metal in CTH of FAL, revealing that introduction of a second metal regulated the electronic structure of Cu species, leading to variation in product selectivity [86]. Li et al. controlled the amount of oxygen vacancies in Co-Al spinel synthesized via the solution combustion method and confirmed that an oxygen vacancy-rich catalyst exhibited a superior MF yield of over 97% [87]. Zhang et al. explored CTH of FAL using a model of a Ni single-atom catalyst with frustrated Lewis pairs, demonstrating that dissociation of isopropanol was facilitated for FAL hydrogenation by the presence of the frustrated Lewis pair [88].

Beyond the experimental and theoretical studies mentioned above, extensive research has been conducted on FAL hydrogenation using noble and transition metal catalysts to selectively produce FA, MF, and other valuable chemicals. Researchers have explored various approaches, including employing hydrogen donor molecules instead of gaseous hydrogen and utilizing electro- and photocatalytic systems. Careful catalyst design is crucial in all cases, as well as sophisticated pre- and post-use catalyst analysis to understand structural and chemical changes during reactions. Additionally, further research on optimizing reaction conditions and systems is necessary to enhance catalytic activity, selectivity, and stability.

## 5. Conclusions and Perspectives

### 5.1. Summary and Significance

This review highlights recent advancements in the synthesis and catalytic applications of mesoporous materials for the efficient design of catalysts in FAL hydrogenation. First, various synthesis methods, such as templating methods, for constructing mesoporous structures were introduced, and their impact on catalyst surface area and pore structure was evaluated. The mesoporous structure played a crucial role in enhancing catalytic performance by enabling high metal dispersion and improving reactant accessibility, thereby influencing both activity and selectivity. Subsequently, strategies for achieving highly dispersed Pd-based noble metal catalysts on mesoporous supports were explored, revealing that the particle size and distribution of Pd varied depending on the synthesis method. Additionally, the type of support material significantly influenced product selectivity. In studies on transition-metal-based catalysts (Co, Cu, Ni), mesoporosity and electronic structure were identified as key factors in controlling reaction activity and selectivity, with bimetallic systems demonstrating enhanced catalytic performance due to strong metal–metal interactions between metal species with distinct properties. Investigations on MOF-derived catalysts provided insights into how the composition and structural modifications of MOFs affect catalytic activity. Furthermore, despite significant advancements in FAL hydrogenation, further improvements in catalyst design are essential to achieve higher efficiency, selectivity, and stability. For instance, in-depth studies on catalyst deactivation mechanisms will aid in developing more stable and durable catalysts. Additionally, the interaction between solvents and catalysts plays a key role in determining both catalyst stability and product selectivity, necessitating continued research in this area.

### 5.2. Spent Catalyst Analysis for Better Catalyst Design

While extensive studies have focused on enhancing catalytic activity and selectivity in FAL hydrogenation, research on catalyst stability and long-term performance retention remains relatively insufficient. In particular, systematic analysis of spent catalysts has often been overlooked. The primary causes of catalyst deactivation, such as metal particle sintering, leaching, and support structure collapse, have been suggested, but their actual impact on catalytic performance and underlying mechanisms require further clarification through detailed investigation. Therefore, future studies should actively employ advanced analytical techniques, such as high-resolution TEM, XPS, and operando or in situ XRD, to precisely track the structural and chemical changes in catalysts before and after reactions. Gaining deeper insight into deactivation mechanisms will be crucial in developing design strategies to enhance long-term catalyst stability.

### 5.3. Reducing Use of Gaseous Hydrogen

Minimizing the dependence on external hydrogen is a crucial approach to enhancing the sustainability of FAL hydrogenation. Various strategies, including CTH, aqueous-phase reforming of methanol, and electrocatalytic hydrogenation, have been explored to achieve this goal. CTH utilizes hydrogen donors such as isopropanol or formic acid instead of molecular hydrogen to selectively hydrogenate FAL. In this process, the catalyst must incorporate both metal active sites and Brønsted or Lewis acid sites, with metal–oxide interactions playing a key role. Aqueous-phase reforming of methanol, on the other hand, involves reforming methanol under aqueous and mild conditions to generate in situ hydrogen for FAL hydrogenation. While this method eliminates the need for an external hydrogen source, it requires carefully designed catalysts capable of simultaneously facilitating oxidation and reduction reactions. Additionally, competitive adsorption of reactants, for example, between isopropanol and FAL, should be considered for efficient catalyst design. Electrocatalytic hydrogenation employs electrochemically generated hydrogen at the electrode surface to achieve selective FAL hydrogenation. Although this approach enables precise control over hydrogenation, it introduces challenges in catalyst and reaction system design due to competition with the hydrogen evolution reaction. While these alternative methods pose challenges in catalyst design compared to conventional high-pressure hydrogenation, the development of bimetallic and metal–oxide hybrid catalysts, the use of mesoporous supports, and the optimization of reaction systems can help overcome these limitations. Future research should focus on designing catalysts that minimize external hydrogen use while maintaining high selectivity and catalytic efficiency.

### 5.4. Effect of Solvent on Product Selectivity

The choice of solvent plays a crucial role in determining product selectivity in FAL hydrogenation, primarily by influencing solvent–catalyst interactions and participating directly in the reaction. Solvents can modify the electronic properties of active sites by coordinating with the catalyst surface, thereby altering the reaction pathway. For instance, polar solvents can interact with metal–oxide interfaces, tuning the electron density of metal sites and favoring specific hydrogenation routes. Additionally, in CTH, solvents such as isopropanol or formic acid act as hydrogen donors, directly influencing the hydrogenation process. The ability of a solvent to donate hydrogen or facilitate proton transfer significantly affects the selectivity toward FA or MF formation. Therefore, future research should also focus on systematically investigating solvent–catalyst interactions to optimize reaction conditions and develop solvent–catalyst combinations that enable selective hydrogenation with minimal side reactions.

## Figures and Tables

**Table 1:** Comparison of CTH of FAL over MOF-808, M-MOF-808, and other MOFs.

Entry	Catalyst	T (°C)	Time (h)	Mass Ratio (Cat. ^1^/FAL/IPA)	Conversion (%)	Y_FA_^2^ (%)	S_FA_^3^ (%)	TOF ^4^ (h^–1^)
1	UiO-66	82	2	0.1/1/25	2.3	1.3	65.5	0.26
2	DUT-52	82	2	0.1/1/25	2.1	0.0	0.0	0.0
3	UiO-67	82	2	0.1/1/25	5.2	0.1	1.9	0.02
4	DUT-67	82	2	0.1/1/25	16.4	13.5	82.3	2.0
5	MOF-808	82	2	0.1/1/25	81.3	66.4	81.7	11.6
6	MOF-808	40	24	0.1/0.5/12.5	27.5	25.4	92.3	0.2
7	M-MOF-808	82	2	0.1/1/25	89.3	79.1	88.6	15.0
8	M-MOF-808	40	24	0.1/0.5/12.5	96.5	85.5	88.6	2.9

^1^ Catalyst. ^2^ Yield of FA. ^3^ Selectivity of FA. ^4^ Turnover frequency.

## Abbreviations

The following abbreviations are used in this manuscript:

FAL	Furfural
FA	Furfuryl alcohol
FR	Furan
MF	Methylfuran
THF	Tetrahydrofuran
THFA	Tetrahydrofurfuryl alcohol
CPO	Cyclopentanone
MOF	Metal–organic framework
EISA	Evaporation-induced self-assembly
ZIF-67	Zeolitic imidazolate framework-67
XRD	X-ray diffraction
TEM	Transmission electron microscopy
OMC	Ordered mesoporous carbon
BET	Brunauer–Emmett–Teller
XPS	X-ray photoelectron spectroscopy
ICP–OES	Inductively coupled plasma–optical emission spectroscopy
TPD	Temperature-programmed desorption
DRIFT	Diffuse reflectance infrared Fourier transform
HAADF	High-angle annular dark field
EDS	Energy dispersive spectroscopy
CTH	Catalytic transfer hydrogenation
